# Multi-sector perspectives on opportunities to increase WIC enrollment through community healthcare partnerships

**DOI:** 10.3389/frhs.2026.1707744

**Published:** 2026-01-29

**Authors:** Sophia E. Allen, Aidan K. Wright, Taralyn Bielaski, Chelsey R. Canavan, Holly Gaspar, Anna M. Adachi-Mejia

**Affiliations:** 1 Department of Obstetrics & Gynecology, Dartmouth Health, Lebanon, NH, United States; 2 Geisel School of Medicine at Dartmouth, The Dartmouth Institute for Health Policy & Clinical Practice, Lebanon, NH, United States; 3 Geisel School of Medicine, Dartmouth College, Hanover, NH, United States; 4 Department of Population Health, Dartmouth Health, Lebanon, NH, United States; 5 Adachi Labs, LLC, Norwich, VT, United States

**Keywords:** child health, food security, maternal health, nutrition, WIC program

## Abstract

**Introduction:**

Participation in Special Supplemental Nutrition Assistance Program for Women, Infants, & Children (WIC) improves health outcomes for birthing people and their children, including reduced preterm birth and low birth weight, and lower rates of nutritional deficiencies for mothers and children.

**Methods:**

This qualitative descriptive study explored opportunities to increase WIC enrollment in two New England states through community healthcare partnerships. We conducted key informant interviews with a semi-structured interview guide, purposively sampling current WIC participants (*N* = 10), clinical providers and staff (*N* = 17), and WIC staff (*N* = 6). We used a combination of deductive and inductive thematic analysis.

**Results:**

Our study revealed that across multiple perspectives – WIC-eligible participants, healthcare providers, clinical staff, and WIC staff – respondents were supportive of increasing WIC enrollment through community healthcare partnerships. Individual-level barriers included limited or inaccurate understanding of eligibility and benefits and perceived stigma, while organizational-level barriers included inconsistent and inefficient integration of WIC referral in clinical settings, lack of clarity about healthcare and WIC staff roles, and scheduling, communication, and other logistical challenges. Facilitators included trusted relationships with healthcare providers and WIC staff, consistent messaging about WIC benefits, and direct assistance with WIC enrollment. Participants advocated for enhancing patient and provider awareness of and education on WIC services, automating the integration of WIC discussions into clinical workflows, and strengthening coordination between WIC and healthcare organizations.

**Discussion:**

Across participant groups, we identified broad support for improving WIC engagement through community-healthcare partnerships. Through analysis of multi-sector perspectives organized by socio-ecological model domains, our results highlight systemic gaps and corresponding opportunities to improve awareness of WIC services and streamline WIC referrals through healthcare-based interventions at the organizational and community levels.

## 1 Introduction

Despite strong evidence that the Special Supplemental Nutrition Program for Women, Infants, and Children (WIC) is associated with improved maternal and child health outcomes, only about half (54%) of eligible families have enrolled nationwide (1, 2). WIC, a food and nutrition assistance program under the United States Department of Agriculture (USDA), provides nutrition assistance, education, and formula and breastfeeding support to income-eligible pregnant women and up to six months postpartum, breastfeeding women up to their child's first birthday, and children under age five (3, 4). Participation in WIC reduces the risk of preterm birth and low-birth-weight infants (5); increases the likelihood that children receive regular medical care and immunizations, and improves early childhood cognitive development and school readiness (6).

WIC directly addresses food insecurity, a critical social determinant of maternal and child health (7, 8). During pregnancy, food insecurity is linked to poor maternal outcomes, including gestational diabetes, iron deficiency anemia, pregnancy-induced hypertension, and perinatal depression and anxiety (9, 10). Poor birth outcomes, such as low birth weight, preterm birth, and increased risk of congenital disabilities such as spina bifida, have also been linked to food insecurity and nutritional deficiencies (9, 11). Food insecurity among children is associated with poor growth and immune system functioning, and the associated stresses from living in a food-insecure household may contribute to poor mental health outcomes (12).

To help address relatively low WIC coverage rates in our area, we examined barriers and facilitators to WIC engagement at a rural academic medical center serving New Hampshire and Vermont across SEM domains. Despite their geographic proximity, WIC coverage rates in 2024 were significantly lower in NH (54%) compared to neighboring VT (72%) (13). In 2021–2022, our health system integrated universal social determinants of health (SDOH) screening and clinic-specific referral structures for positive food insecurity screening. This context provided a natural opportunity to build clinical workflows and community partnerships for increasing referrals for eligible families to WIC services. Among our patient population, approximately 5.3% of pediatric patients and 10.2% of obstetrics patients currently screen positive for food insecurity (unpublished internal data), as measured by the Hunger Vital Signs questionnaire (14). Due to our rural geography, many patients may live 20 or more miles from the nearest full-service grocery store. Additionally, food pantries have limited hours, and food options are not always tailored to address the nutritional needs of women and children. Compounded by limited public transportation availability, accessing existing food supports may not be feasible for all patients. Unfortunately, food insecurity rates will likely continue to increase due to recent eligibility cuts to federal food and nutrition assistance programs, making it especially relevant to address challenges associated with accessing WIC services.

Among the often-cited barriers to using WIC, prior studies have identified obstacles at multiple levels, including administrative, healthcare, policy, and individual factors (12, 15–17). Administrative and healthcare system barriers include difficulty navigating enrollment and recertification processes, burdensome paperwork, limited appointment availability, long wait times at WIC clinics, and challenges coordinating WIC visits with other healthcare or social services (16, 17). Policy-level barriers include inflexibility of approved food packages to accommodate dietary preferences or allergies and eligibility or recertification requirements that can disrupt continued participation (12, 16, 17). Individual-level barriers include lack of knowledge about how to apply, misconceptions about eligibility and benefits, and perceived stigma from social networks, healthcare workers, or at the grocery store (12, 16, 17). Although WIC covers children up to age five, national data show a decline in participation as children age, highlighting a persistent and preventable gap in program retention (2).

**Figure 1:**
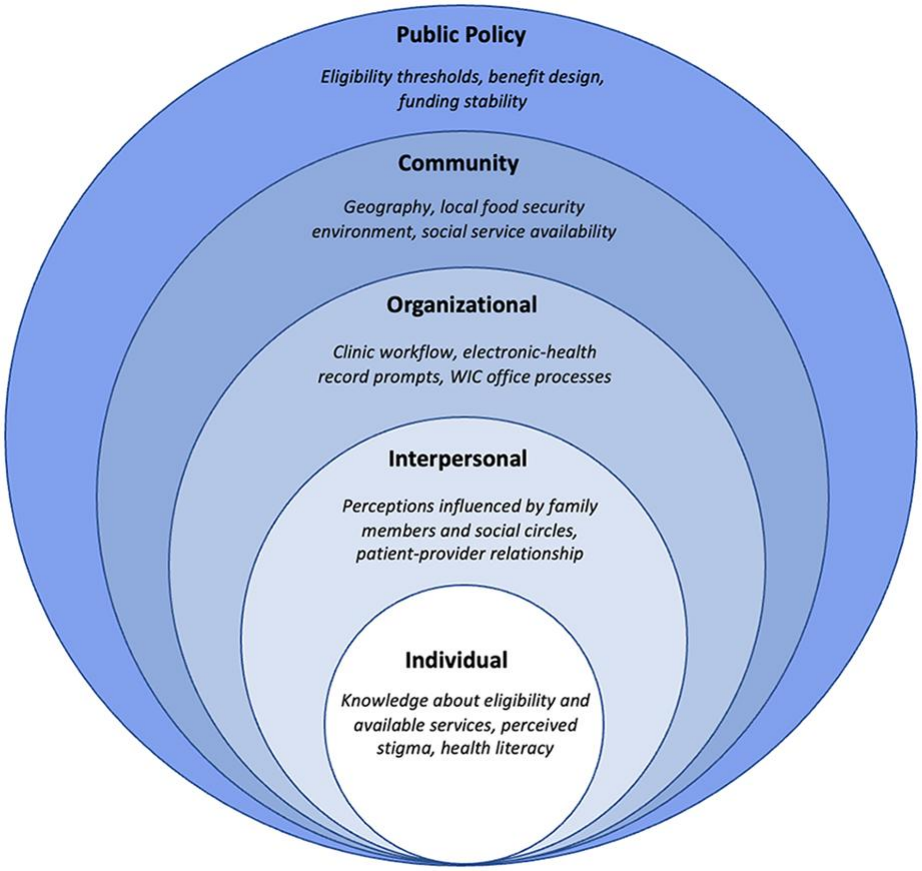
Documented factors influencing WIC engagement organized by socio-ecological model (SEM) domains.

Given the multi-level, overlapping factors that influence WIC participation, we used the Socio-Ecological Model (SEM) to guide our examination of barriers and facilitators (18) (Figure 1). Using the SEM to frame our study enables systematic identification of leverage points to improve WIC participation in a rural New England setting. Prior studies have explored limitations to WIC participation (12, 15–17) separately from the perspectives of WIC-eligible patients, clinical staff, and WIC staff. Beyond documenting barriers and facilitators, our analysis integrates these different perspectives to identify system-level sources of misalignment between clinical workflows, administrative processes, and WIC education at a single rural healthcare system. Our findings are intended to inform the development of healthcare-based interventions and efforts to strengthen community partnerships in our rural region, offering insight into how to utilize multi-sector perspectives to increase WIC engagement in similar rural settings.

## 2 Methods

### 2.1 Design

As part of an effort to increase WIC engagement via healthcare-based interventions, we conducted a qualitative descriptive evaluation using semi-structured interviews, which examined multi-sector perspectives on leveraging community healthcare partnerships to increase WIC enrollment across SEM domains. The Dartmouth Health Institutional Review Board determined that this evaluation was not human subjects research. We followed the Consolidated Criteria for Reporting Qualitative Research guidelines (19).

### 2.2 Participants and setting

We purposively recruited WIC-eligible participants (*n* = 10) from a regional NH WIC agency site via flyers; healthcare providers (*n* = 8) and staff (*n* = 9) from pediatrics (*n* = 3), OB-GYN (*n* = 10), and family medicine departments (*n* = 4; all part of one health system) via email; and six NH and VT WIC program staff via email. Of note, we sought to understand why eligible patients chose not to enroll in WIC and were referred to four patients who fit this category; however, these individuals all declined to be interviewed.

### 2.3 Data collection

Our project team held several meetings to develop a semi-structured interview guide (see Supplemental Materials), which included participant-specific questions on challenges and opportunities for increasing WIC engagement through healthcare referrals. AKW and SEA scheduled and conducted interviews (median length of 33 min) between February and April 2024 via Zoom or Webex videoconferencing. We received verbal consent from all participants and audio-recorded and professionally transcribed all dialogue. WIC participants who completed interviews received a $25 gift card; healthcare and WIC staff were not compensated for their participation.

### 2.4 Data management and analysis

We used a combination of deductive and inductive thematic analysis to code interviews (20). Deductively, we derived codes from the interview guide informed by prior literature on barriers and facilitators to WIC enrollment and retention, and our primary aim of exploring the potential of community healthcare partnerships to increase WIC engagement. Within these codes, we constructed subcodes that emerged inductively from participant interviews.

Two research team members (SEA and AKW) manually coded each de-identified transcript independently and assigned codes to a third of the transcripts at a time. To code, we used the commenting feature in Microsoft Word and then merged/ organized all codes and corresponding quotations in an Excel spreadsheet (21). A third team member (TB) validated the other two coders' approach by carefully reviewing all transcripts and adding notes, questions, or additional codes to the coding schema where necessary. All research team members read all transcripts and completed analytic memos to facilitate iterative interpretation of the data. We met three times throughout the coding process to resolve coding discrepancies and revise the codebook as new insights emerged. We also met twice with the full author team (AMAM, CRC, HG) to review our approach and validate ongoing findings.

### 2.5 Reflexivity

All authors identify as female, and three are mothers. The senior author has a doctorate degree (AMAM), the second author is a first-year medical student (AKW), and all other authors have master's degrees (SEA, TB, CRC). While none of the authors are clinical providers, all had comprehensive knowledge of WIC program eligibility and services, which may have influenced data collection, analysis, and interpretation. We all completed positionality notes and reflected on saturation throughout the analysis process, per thematic analysis guidelines (22).

## 3 Results

We conducted interviews with WIC-eligible participants, clinical providers and resource/ allied support clinical staff, and WIC staff (see Table 1 for sample characteristics; due to the small sample size and to protect participant confidentiality, we did not collect demographic information). We organized themes deductively by health system barriers and facilitators to WIC engagement, grouped by participant type and corresponding SEM domain (Tables 2, 3).

**Table 1:** Characteristics of interview participants (*n* = 33).

Perspective	Role/Description	*N*
WIC Participants (*n* = 10)	WIC enrollment status	

	Currently enrolled	8
	Pursing enrollment	2
	Timing of WIC enrollment	
	During pregnancy	5
	During postpartum period	5
	Previous WIC enrollment	
	First time participant	7
	Prior WIC participation	3
	Method of learning about WIC	
	Through healthcare provider/community referral	2
	Through friends/family	8
Clinical Providers (*n* = 8)	Professional title	

	Certified Nurse Midwife (CNM)	2
	Pediatrician (MD)	3
	Advanced Practice Registered Nurse (RN)	1
	Registered Nurse (RN)/Nurse Manager	2
Allied-Support and Resource Staff (*n* = 9)	Professional title	

	Community Health Worker (CHW)	4
	Social Worker (SW)	1
	Dietician	1
	Case Manager/Resource Specialist	3
WIC Staff (*n* = 6)	New Hampshire (NH)	

	Program Assistant	1
	Program Manager	1
	Breastfeeding/Lactation Coordinator	1
	Vermont (VT)	

	WIC Director	2
	Program Assistant	1

**Table 2:** Barriers to WIC engagement.

Perspective	Theme – Barriers	Quote	SEM domain
WIC Participants	Misconceptions or confusion about WIC eligibility, program details, or substitutions	*“I’m not even gonna bother”*	Individual/Interpersonal
	Lack of capacity to engage with WIC	*“Typical parent overwhelm”*	Individual/Interpersonal
	Not feeling represented by the WIC program	*“This is just not going to work for me”*	Organizational/Policy
	Perceived stigma of using WIC benefits	*“I felt judged”*	Interpersonal
Clinical Providers & Staff	Perception of patients’ experience of stigma	*“There's still social stigma”*	Interpersonal
	Knowledge gaps and feelings about knowledge gaps	*“We’ve never really been educated”*	Organizational
	WIC discussions are not integrated into clinical workflows	*“I don't know unless they tell me”*	Organizational
	Limited time during visits	*“WIC doesn't always come up”*	Interpersonal/Organizational
WIC Staff	Misconceptions about WIC services	*“There are huge gaps in understanding”*	Individual/Interpersonal/Policy
	Capacity and other ambiguous barriers	*“There was no definitive reason”*	Individual/Community

**Table 3:** Facilitators to WIC engagement.

Perspective	Theme – Facilitators	Quote	SEM domain
WIC Participants	Kind and responsive staff who care about families	*“Really lovely to work with”*	Organizational
	Convenient to enroll and use WIC benefits	*“It's pretty seamless”*	Organizational/Community
	Specific supports for becoming a parent	*“A bunch of reliefs”*	Organizational/Policy
Clinical Providers & Staff	Framing WIC as a resource for the child	*“Help taking care of your baby”*	Interpersonal
	Referral to community health workers (CHWs) and social workers	*“I really try to connect them”*	Organizational
	Continuity of care and integration of WIC conversations	*“It's something I talk to all of my patients about”*	Interpersonal
	Awareness of how nutrition impacts health outcomes	*“Being cognizant of social needs”*	Interpersonal/Organizational
WIC Staff	WIC is convenient and flexible	*“We try to make it easy”*	Organizational

### 3.1 WIC participants - barriers to WIC enrollment and retention

#### 3.1.1 “I’m not even gonna bother”: misconceptions or confusion about WIC eligibility, program details, or substitutions

Participants described various misconceptions about WIC products and services. For example, one participant was unaware that she was eligible for WIC while pregnant, assuming she was only eligible after birth. Another participant did not know she could receive WIC benefits while breastfeeding. Two participants were unaware that certain WIC food package items could be substituted; one mother did not request lactose-free milk (despite it being an option) because a friend told her not to bother “jumping through the hoops.” Another participant, who was considering disenrolling from WIC, thought she was taking away from someone who “needed WIC more” and felt guilty about not using her full benefits. Despite changes in WIC benefit utilization designed to address barriers in the WIC shopping experience (e.g., the shift from vouchers to an electronic benefits card and the WIC shopper app), one participant thought that you still had to ring up WIC purchases separately, which contributed to her feeling “anxious while checking out.”

#### 3.1.2 “Typical parent overwhelm”: lack of capacity to engage with WIC

Participants talked about many new responsibilities associated with pregnancy and parenting, and even those who had previously used WIC spoke about barriers to re-enrolling. For example, one participant previously enrolled in WIC before moving to NH said, “WIC, unfortunately, got put on the back burner and kinda almost got forgotten about…I had so many other things to put first.” When asked if her healthcare providers could help support her in getting connected to WIC, another participant shared that she already felt overloaded with information in medical settings and wanted more “hands-on” help to understand the WIC program instead of “pamphlets that get lost or end up in the trash.”

#### 3.1.3 “This is just not going to work out for me”: not feeling represented by the WIC program

Participants shared several functional barriers, including WIC being too specialized, SNAP being “less specific” and therefore more appealing, feeling “patronized” by WIC covering only certain brands, and WIC products not being stocked where participants normally shopped (e.g., Walmart). One participant did not use her full WIC benefits as she lacked food preparation and cooking skills:

“I feel like what ends up happening is that people are either really resourceful with it [their WIC benefits], and they know exactly what to get the healthy stuff, they know exactly how to find it, they know exactly how to prep things with it. And then other people like myself don't know how to be resourceful with it…I didn't grow up in a mommy cooks dinner type of household.”

Another common sentiment was that the “need for WIC lessens,” meaning that specific perks of the WIC program (namely breastfeeding support and formula coverage) were not replaced with equally helpful support as children grew.

#### 3.1.4 “I felt judged”: perceived stigma of using WIC benefits

While most did not explicitly speak about stigma related to needing or using their WIC benefits, several people alluded to feeling uncomfortable or anxious about using their benefits (e.g., when asking for help to find the right WIC product, waiting in the checkout line, or having to explain WIC benefits to grocery clerks). However, one person (a foster parent) talked about stigma unprompted throughout her interview. She felt that using WIC garnered “a lot of attention,” adding to her feeling of “self-consciousness” because her foster child does not look like her. This participant expressed that she “felt judged,” but was working towards feeling “more confident in using [her] WIC card” and more “deserving of [her] benefits.” While some participants described negative social perceptions, pressures, and interactions associated with checking out food, one participant stated that she has never felt stigmatized for using WIC.

### 3.2 WIC participants - facilitators to WIC enrollment and retention

#### 3.2.1 “Really lovely to work with”: kind and responsive staff who care about families

Almost everyone expressed gratitude for the WIC staff, describing them as “kind,” “friendly,” “thoughtful,” “totally helpful,” “understanding,” and overall “good people.” Participants valued that WIC staff checked in on them and were excited to hear about their children's development. For instance, one participant was effusive about how helpful the staff were in getting her the right formula during the formula shortage, and another was impressed by the staff's responsiveness when she called with questions from the grocery store. Even when participants identified areas for improvement in the WIC program, no one explicitly attributed their frustrations to WIC staff.

#### 3.2.2 “It's pretty seamless”: convenient to enroll and use WIC benefits

Once connected, most participants described the program as convenient or easy to use. They appreciated clear eligibility requirements (e.g., knowing they were eligible through Medicaid or SNAP benefit use), ease of signing up for and attending appointments (e.g., enrolling at a local church), and having a provider who consistently checked in about household nutrition and WIC benefit use. One participant appreciated being able to sign up for WIC while at a different social service appointment, which prevented “multiple trips to different locations.” Another participant shared that WIC was basically “placed into [her] lap at the hospital,” when a social worker walked her through the application. Another participant was encouraged by the level of support WIC offers, emphasizing that it was “not that difficult to go to an appointment once in a while.”

#### 3.2.3 “A bunch of reliefs”: specific supports for becoming a parent

Four participants used the word “relief” when describing aspects of the WIC program, including financial assistance, breastfeeding and lactation guidance, nutrition education, and general support for becoming a parent. Some kept their praise general, including, “WIC provides healthy things that you really need,” or, “They provide whatever it is that you might need…like every mom needs help in different ways.” Others went into more detail:

“The best thing at the time that I was breastfeeding was honestly getting formula because I had a COVID baby, and she was taking formula, but the cans are like $19 a can. So it was super expensive, and I didn't have full coverage for cans with EBT. And I knew that I could always depend on WIC to provide that. I didn't have to worry about spending money for my own food to provide for my daughter.”

Notably, many focused on the support that they received for breastfeeding and formula, elevating the specific importance of lactation support.

### 3.3 Clinical providers and staff - barriers to discussing WIC

#### 3.3.1 “There's still social stigma”: perception of patients’ experience of stigma

Several clinical staff members focused on perceived stigma among patients throughout their interviews. A CHW mentioned that her patients sometimes had some “nervousness” around disclosing their financial status, and a CNM hypothesized that some of her patients “[withheld] information” when she asked about their needs. Of note, clinical staff did not elaborate on whether they thought patients' experiences of stigma made them more or less likely to talk about WIC, only that they assumed that patients felt stigmatized based on cues from clinical encounters.

#### 3.3.2 “We’ve never really been educated”: knowledge gaps and feelings about knowledge gaps

Clinical staff discussed knowledge gaps both objectively and subjectively. Some providers emphasized that their training did not include information on WIC; a CNM said, “I don't even know how to do the paperwork…no one has ever shown me, it's always kind of in someone else's job description.” This provider went on to say she felt some “guilt” and “awkwardness” around speaking about WIC because she felt undereducated. Another OB-GYN nurse “felt embarrassed that [she didn't] know more,” contributing to her discomfort with having “specific conversations [about WIC] with patients.” A CHW whose job description involves discussing social needs with patients felt she needed “more information about WIC and additional benefits because [she] knows the basics but not the details.”

Notably, some providers were unaware that being on Medicaid or Medicare insurance automatically qualified an individual for WIC. Many providers were more familiar with local resources for food insecurity than national public assistance programs such as WIC or SNAP. A nurse supervisor summarized the knowledge of her staff regarding WIC as follows: “Providers don't know enough about WIC to set expectations for the patient.” Despite these limitations, providers across various roles wanted to know more about WIC and were eager for tools to help them discuss WIC with patients.

#### 3.3.3 “I don't know unless they tell me”: WIC discussions are not integrated into the clinical workflow

Regardless of standard screening practices, providers commented that WIC program-specific discussions were not integrated into clinical workflows. An OB-GYN physician acknowledged that she did not often ask about WIC but suspected that many patients “may have some degree of food insecurity that's not obvious.” Other providers mentioned that WIC status is not systematically documented, making it difficult to discuss food support. Many thought it would be helpful to have clinical flags in the electronic health record for WIC eligibility and systematically track WIC status. A pediatric physician concluded:

“On the medical end, it's probably more just having a consistent mechanism with the prompt because there are a lot of things that we have to ask families about…and if there's not the structure there for something like WIC, that's more likely to not happen because it's not emergent.”

Two providers (both OB-GYN nurse midwives) were adamant about not looking at patients' insurance status; in one provider's words, she “[wanted] to come to each interaction revenue-neutral” to not “bias any of the things that [she's] counseling the patient on.” The other provider felt that only social workers and case managers should look at insurance and that other providers should practice from a “non-insurance-based stand.” Both providers still mentioned WIC on an ad-hoc basis if patients brought up financial or food insecurity themselves. In contrast, a case manager said she immediately mentions WIC and SNAP if someone has Medicaid insurance and goes through the program details, addressing any questions or misconceptions on the spot.

#### 3.3.4 “WIC doesn't always come up”: limited time during visits

Most clinical staff mentioned a lack of time and other priorities during a visit as a reason that social needs are overlooked. One CHW noted:

“It's not a conversation that comes up frequently with the providers. Although the information is there and providers can review the screening [referring to a social determinants of health screener], they're trying to fit so much into a short period of time and don't always get to food insecurity..”

A physician mentioned that discussing WIC might not be appropriate for providers who deal with high-risk cases (such as maternal-fetal medicine), as these providers must address urgent medical needs. Another physician relied on social workers to consult about social needs because she does not have time to “read through every note in mom's chart.” A CHW and a social worker noted that WIC offices were sometimes unresponsive or slow to respond when they called to enroll patients, resulting in “gaps in care.”

### 3.4 Clinical providers and staff - facilitators to discussing WIC

#### 3.4.1 “Help taking care of your baby”: framing WIC as a resource for the child

Providers discussed different ways of framing WIC and shared anecdotes of positive patient interactions. One CNM talked about WIC simply as a resource for “taking care of your baby,” which she felt “[resonated] more than just a resource for taking care of themselves.” A CHW and a social worker worked to normalize discussions about food and other needs, starting with these conversations to build a trusting relationship. Both emphasized framing supports as “opportunities” rather than “something offered based on a perceived need or deficit.”

Resource/allied-support professionals took the time to debunk myths and misconceptions about WIC, including educating patients on the duration of eligibility or how to request food item substitutions. A case manager often told patients that she would refer them even if they weren't on Medicaid insurance, pointing out that checking if they qualified was worth it.

#### 3.4.2 “I really try to connect them”: referral to CHWs and social workers

Referral practices differed by way of provider and clinic. All but one provider referred patients to resource specialists, CHWs, or social workers, and were confident that this was the most efficient way to support patients and ensure appropriate care coordination. A CNM emphasized that patients need “help with paperwork, to make sure everything goes through, and someone to follow up.”

Resource/allied-support professionals agreed that their roles were well-suited to facilitating WIC enrollment but felt that a warm handoff from providers facilitated a stronger connection with the patient. Several mentioned that guiding patients toward online referrals was “pretty seamless” and connected with specific WIC staff to facilitate quicker referrals.

#### 3.4.3 “It's something I talk to all of my patients about”: continuity of care and integration of WIC conversations

Several pediatric providers mentioned that WIC often comes up in well-child visits, as they are mandated to discuss children's nutrition and eating habits. Two pediatricians always mentioned WIC as “part of clinical conversation” during well-care visits, a structure that differed from obstetric workflows. Resource/allied-support staff also initiated consistent conversations about food and nutrition during their initial patient assessments. Even providers and clinical staff who were very comfortable discussing WIC emphasized the need for a better way to capture WIC eligibility and enrollment status.

#### 3.4.4 “Being cognizant of social needs”: awareness of how nutrition impacts health outcomes

Clinical staff were supportive of normalizing conversations about social needs and addressing barriers within clinical settings:

“It's often maybe someone doesn't have a phone, or they're not able to get to their WIC appointment, they're not able to maybe get their information signed by the doctor if that's needed. In any of those cases, that's where I would try to assist, try to eliminate those barriers so that they can get what they need.” (resource specialist)

Some specifically outlined the importance of nutrition on health during pregnancy and child development. When asked about strategies to improve healthcare response to food insecurity and other social needs, clinical staff mentioned screening patients multiple times throughout pregnancy and childhood visits and leveraging the electronic medical record system to automate referrals.

### 3.5 WIC staff (VT and NH) - barriers to WIC engagement

#### 3.5.1 “There are huge gaps in understanding”: misconceptions about WIC services

WIC staff all mentioned a lack of standardized education about WIC among patients and providers alike. Staff members echoed patient-reported misunderstandings, including the lack of understanding that eligibility begins during pregnancy and continues throughout breastfeeding, as opposed to only the child being covered after birth. An NH breastfeeding coordinator added that changing program requirements (e.g., “patients no longer have to show proof of pregnancy,” and the switch back to in-person WIC certification visits after virtual visits during the pandemic) has led to lingering misconceptions. An NH program assistant attributed most of this confusion to most people hearing about WIC through a family member or friend who may not have accurate information, along with lack of coordinated marketing to spread awareness about the full scope of WIC or program changes.

#### 3.5.2 “There was no definitive reason”: capacity and other ambiguous barriers

WIC staff cited client unresponsiveness and other ambiguous capacity barriers as major challenges to enrollment and retention in the WIC program. An NH program assistant described having the same conversations repeatedly with people who waited to connect with WIC, despite knowing they were eligible. She added that people generally did not explain why they waited, except to state that WIC was not a priority. Staff members from NH and VT also cited transportation as an enduring challenge to participation in the WIC program and full use of benefits. The VT WIC director concluded that, despite wide-ranging services, WIC will “always be supplemental,” and cannot fulfill all unmet needs, deterring some individuals from enrolling.

### 3.6 WIC staff (VT and NH) - facilitators to WIC engagement

#### 3.6.1 “We try to make it easy”: WIC is convenient and flexible

WIC staff emphasized the importance of building trusting relationships with program participants and maintaining frequent contact. To accommodate flexibility for in-person appointments, WIC staff offered early morning or weekend appointments, as well as the option to send a child to their appointment with a relative or friend.

WIC staff also mentioned ongoing efforts to increase options for enrolling in WIC (i.e., online portal, phone call, or Medicaid referral) and maintaining timely communication with both eligible and enrolled clients. An NH program manager appreciated the Medicaid referral system because she felt it was “easier” to put the onus on WIC staff to make the connection. An NH program assistant was proud of how her team had “streamlined the process” of recertification, adding that she “[didn't] really know how much simpler it could be, aside from picking people up for their appointments.” A VT WIC program manager summarized her thoughts on her team's commitment to customer service:

“We do a lot of work also around retention. There's a lot of outreach to make sure people stay on the program. So it's not just like, “Oh, you're due for an appointment, a letter in the mail, hope that works out for you.” We do follow-up calls, text messages, we will try everything to connect to make sure you're still able to continue to access the program and take that off people's plates.”

All WIC staff emphasized the importance of outreach and program retention efforts, as well as proactive efforts to improve engagement.

## 4 Discussion

Overall, participants across groups described WIC as providing “a bunch of reliefs” for families, highlighting the program's potential to improve financial, nutritional, and educational support during pregnancy and early childhood. To interpret our findings, we apply the Socio-ecological Model (SEM), which emphasizes how influences at the individual, interpersonal, organizational, community, and policy levels influence WIC engagement. While all groups were enthusiastic about WIC participation, our findings suggest that organizational improvements are essential for improving engagement in the program.

At the individual and interpersonal levels, awareness and framing of WIC emerged as central drivers of engagement. WIC participants expressed overwhelmingly positive feelings about the support they received through the program; clinical staff appreciated the opportunity to offer their patients resources; and WIC staff took pride in their responsiveness and commitment to supporting families. All groups wanted WIC to be presented as a welcoming, easy-to-use support system, a financial relief, a safety net, an educational opportunity, and ultimately a routine part of parenting. Notably, most WIC participants we interviewed learned about their eligibility for the program from their peers, which is encouraging but also raises the potential for misconceptions about WIC services to perpetuate.

At the organizational level, we found that clinical providers had uneven, minimal, or no awareness of WIC's services and eligibility requirements, despite recognizing the importance of connecting patients to community-based resources. A few clinical staff had no understanding of WIC, most had knowledge that WIC exists but a limited or inaccurate understanding of services, and only a few had a complete understanding of WIC's current services. Providers struggled to prioritize discussions about WIC, citing limited time, competing demands, lack of education, the absence of dedicated staff to coordinate workflows, and discomfort with conversations about social needs. Staff trained in resource navigation, specifically community health workers (CHWs), social workers, and nurses, felt most comfortable discussing WIC and were perceived by patients as most helpful in connecting to WIC. These findings align with evidence demonstrating that social-needs assessments and referral practices for food and nutrition insecurity are not consistently integrated into routine care, despite the widening availability of screening tools (23) and documented acceptance of screening for social needs among clinical providers and staff (24).

All groups expressed frustration about perceived unresponsiveness, underscoring systemic communication challenges. For example, some patients struggled to discuss their needs due to limited appointment time, while clinical staff worried that patients downplayed or omitted discussion of food insecurity. WIC staff expressed frustration that patients were not more responsive to outreach and that clinical staff were either unable or unwilling to prioritize WIC-specific education, despite their influence over patients' health behaviors. These perspectives coalesced around the need to improve outreach and partnerships between WIC and healthcare organizations, including efforts to develop clearer guidelines for clinical staff roles in facilitating WIC referrals and to enhance provider and patient education about WIC services. Several participants recalled knowing they were eligible for WIC but not having the time or resources to enroll until the postpartum period, underscoring the need to streamline WIC referral processes, particularly during prenatal care. These communication challenges reflected organizational misalignments, rather than any group's lack of interest in strengthening WIC referral processes.

Participants described multiple opportunities to strengthen partnerships between WIC and healthcare systems. Echoing existing literature, respondents endorsed organizational-level strategies such as co-locating WIC clinics and primary care clinics (15), increasing provider education and screening (12), and automating referral processes via electronic health records (25). Recognizing limited resources and staff capacity, most focused on increasing education about WIC and streamlining referrals to WIC as feasible first steps to increase WIC enrollment. Clinical staff also suggested repeat screening throughout pregnancy and during childhood visits, as well as integrating more accessible systems for tracking WIC eligibility and enrollment status. Priorities for strengthening outreach and partnerships between healthcare and WIC included: building awareness among patients, providers, and clinical staff through sustained education; standardizing WIC screening and referral processes; and tracking WIC enrollment and engagement over time to inform quality improvement.

Given the dynamic national policy environment surrounding WIC, including continued threats to public food assistance eligibility and funding, growing involvement and advocacy at the healthcare and community levels will be increasingly important for streamlining referrals and expanding education. Future research should explore the extent to which these policy shifts shape WIC awareness, eligibility, and participation over time. Additionally, as recognition of the health benefits of WIC participation expands, ongoing workforce shortages and clinic capacity constraints may intensify organizational-level barriers, underscoring the need to proactively increase clinical and WIC staffing infrastructure to meet rising demand.

Our results are limited in transferability as they are based on one health system with a modest sample size; however, the health system serves two states (NH and VT) with differing public and social service infrastructures, as well as both rural and micropolitan geographies. The omission of WIC participant demographic data is warranted due to our small sample size, but limits discussion of geographic, social, and economic factors that may be associated with disparities in regional WIC participation. Additionally, we were unable to interview eligible individuals who chose not to enroll in WIC, raising the risk that future interventions may not address comprehensive barriers to program participation.

Applying the SEM framework (18), our findings demonstrate that barriers to WIC engagement at any single level were amplified by constraints at outer levels. For example, individual knowledge gaps were reinforced by interpersonal stigma and compounded by the absence of supportive clinical workflows. Consequently, interventions must be multifaceted, including provider and patient education (individual and interpersonal) together with organizational processes and workflows (organizational). Implementation science approaches are well-suited to evaluate the feasibility, acceptability, and sustainability of future healthcare-facilitated educational and referral interventions for WIC enrollment and retention rates among perinatal and pediatric patients, and to assess their applicability to other geographic and clinical settings.

## Data Availability

The datasets presented in this article are not readily available; to protect the privacy of our participants, we will only make de-identified data available upon reasonable request. Requests for data may be sent to the corresponding author.
