# Supplementary material for: Multi-sector perspectives on opportunities to increase WIC enrollment through community healthcare partnerships
Source: Front Health Serv. 2026 Jan 29;6:1707744. doi: 10.3389/frhs.2026.1707744 (PMC12894314; doi:10.3389/frhs.2026.1707744)
Supplement: Supplementary file 1 [file Table1.docx]

**Interview Guide: WIC Participants**

*Thank you for agreeing to chat with me today. As we mentioned when we reached out, we are trying to develop community-healthcare partnerships to improve participation in the WIC Program [a federally funded program that supports the nutritional needs of women, infants, and children.].  The hope from these interviews is to get a better sense of what is working, and where there are challenges and opportunities for improvement. In particular, we know that many individuals who are eligible for WIC don’t sign up, and even among those who do sign up, many do not continue to attend appointments to receive benefits for their full eligibility period or make full use of their food benefits. Given that you [are someone who has engaged with WIC or may potentially consider/be eligible for WIC] we are hoping you will share your expertise, experiences, and perspectives with us. This interview is completely confidential – none of your responses will be linked to your name. We can also skip any questions you prefer not to answer. Is now still a good time for you?  And do you mind if I record this conversation?*

*[if yes to recording, should restate at beginning of recording that you are recording, e.g. “Thanks for agreeing to have this conversation recorded.”*

*Groups to include:*

- *Pregnant women/moms of kids <5 years old with WIC experience*
- *Pregnant women/moms of kids <5 years old without WIC experience*
- *Caregivers with WIC- eligible children <5 years old with WIC experience*
- *Caregivers with WIC- eligible children <5 years old without WIC experience*

1. Thank you for agreeing to talk to me today about WIC. WIC is the common name for the Special Supplemental Nutrition Program for Women, Infants, and Children. Can you tell me what comes to mind when I mention WIC?
   1. Are you already familiar with the WIC Program? If so, can you tell me how you learned about WIC?
   2. Can you tell me what you know about the services WIC provides?
2. Do you have experience with the NH WIC Program, either for yourself or for a child?
   1. For yourself?
   2. For a child?
   3. How many years of experience?
3. [If yes to #2] Can you walk me through your experience of trying to enroll or explore WIC to potentially signing up, receiving nutrition education, and using the food benefits at the store, etc.?
   1. What was the experience like?
   2. What made it easy? What made it hard?
   3. What motivated you to sign up? Did anyone encourage you?
4. [If yes to #2] What has it been like participating in the WIC Program?
   1. What makes you feel good or not good about participating in the WIC Program?
   2. Do you have any concerns receiving WIC services, or anything you don’t feel good about?
   3. Do you think you will continue to participate in WIC until your child turns 5 years old? (Did you know your child can participate until age 5?)
      1. Why might you continue? Why might you not continue?
5. [If no to #2] Can you tell me about your reasons for not participating in the WIC Program?
   1. Do you know if you/your child qualifies for WIC? How would you check if you/your child are eligible?
   2. What would encourage you to consider enrolling and/or learning more about it?
   3. What would discourage you?
   4. Do you have any challenges with using cell phone or computers to make appointments or complete form?
6. Sometimes individuals who have enrolled in WIC don’t end up using all their food benefits each month. Why do you think that happens?
   1. (If using WIC, can probe if they have ever had that experience)
   2. What would make you more likely to use all your food benefits each month?
7. Have you ever used any other food support or assistance programs, like SNAP? [If so, how is that going?]
   1. Did your use of other supports make you more or less likely to look into WIC?
8. Is there anything your healthcare provider or office could do to support you or assist you with enrolling in the WIC Program?
   1. Is there anything else that could improve your experience?
   2. What are healthcare providers’ roles in discussing food and other needs with patients?
   3. Who should have these conversations with patients?
9. Is there anything you would recommend to other women or caregivers of young children who might be able to benefit from WIC?
   1. What would be helpful for them to know?
10. Is there anything else you would like to share?
11. Would it be okay to reach back out to you in the future?

**Interview Guide: Clinical Providers & Staff**

*Thank you for agreeing to chat with me today. As we mentioned when we reached out, we are trying to develop community-healthcare partnerships to improve participation in the WIC Program [a federally funded program that supports the nutritional needs of women, infants, and children.]. The hope from these interviews is to get a better sense of what is working, and where there are challenges and opportunities for improvement. We know that many individuals who are eligible for WIC don’t sign up, and even among those who do sign up, many do not continue to attend appointments to receive benefits for their full eligibility period or make full use of their food benefits. Given that you are a healthcare provider who may encounter patients who are WIC-eligible, we are hoping you will share your expertise, experiences, and perspectives with us. This interview is completely confidential – none of your responses will be linked to your name. We can also skip any questions you prefer not to answer. Is now still a good time for you?  And do you mind if I record this conversation?*

*[if yes to recording, should restate at the beginning of recording that you are recording, e.g., “Thanks for agreeing to have this conversation recorded.”*

*Healthcare staff include: pediatrics, family medicine, and OB-GYN providers; other clinical staff (e.g., community health workers, social workers, case managers)*

1. What is your role in helping to identify or address food insecurity among pregnant women and children?
   1. Who do you think is best positioned to have these conversations with patients?
2. What kinds of conversations, if any, do you have with individuals about food security and nutrition?
   1. How do these conversations go?
   2. When do you have these conversations?
   3. Are there certain indicators for when to have these conversations?
   4. How do you feel these conversations are received by patients?
   5. Do you feel like these conversations influence patient behavior or nutrition?
3. When you hear ‘WIC,’ what comes to mind? *(If there are knowledge gaps, explain eligibility and use.)*
4. What do you see as your role in discussing the WIC Program with patients?
   1. Can you tell us about when/how/if you talk to your patients about WIC?
      1. What do/would you say about WIC to patients?
      2. What WIC services do you typically bring up?
   2. What makes it easy to have conversations about WIC? What makes it hard?
   3. What, if any, ideas do you have on how to improve conversations about WIC?
   4. Thinking about other support programs besides WIC, such as SNAP, resource centers, food pantries, etc., to what extent are these other programs part of the conversations that you have with patients?
   5. How do you know if your patients are enrolled in WIC? Would it be helpful to know?
      1. How should this information be captured?
5. What is the role of healthcare to support patients engaging with WIC? Is this an important piece to their overall health?
   1. What is your experience incorporating WIC into patients’ care plans?
6. In your experience, what barriers are there to women and children enrolling in NH WIC?
   1. Is there anything that has helped enroll patients?
   2. If resources and time were not a constraint, what changes would you make to facilitate greater enrollment and utilization of WIC benefits among your patients? (*could prompt for DH system*)
7. Often, individuals participating in WIC discontinue as their child gets older.
   1. Can you share any ideas why this may be the case?
   2. Have any of your patients shared anything with you about this?
8. In relationship to screening and referrals to community services, what has your experience been like when sharing patient data across organizations (outside of DH)?
   1. What has worked well?
   2. Where have challenges occurred? How were they navigated?
9. Is there anything else you would like to share?

**Interview Guide: New Hampshire WIC Staff**

*Thank you for agreeing to chat with me today. As we mentioned when we reached out, we are trying to develop community-healthcare partnerships to improve participation in the WIC Program [a federally funded program that supports the nutritional needs of women, infant, and children.].  The hope from these interviews is to get a better sense of what is working, and where there are challenges and opportunities for improvement. In particular, we know that many individuals who are eligible for WIC don’t sign up, and even among those who do sign up, many do not continue to attend appointments to receive benefits for their full eligibility period or make full use of their food benefits. Given your role with WIC, we are hoping you will share your expertise, experiences, and perspectives with us. This interview is completely confidential – none of your responses will be linked to your name. We can also skip any questions you prefer not to answer. Is now still a good time for you?  And do you mind if I record this conversation?*

*[if yes to recording, should restate at beginning of recording that you are recording, e.g. “Thanks for agreeing to have this conversation recorded.”*

*NH WIC clinic/local office personnel*

1. What is your role with WIC? How long have you worked for NH WIC?
2. We’d like to better understand your experiences working with people to enroll in the WIC Program and stay participating through their entire eligibility period.
   1. What works well?
   2. What could make it easier?
   3. What barriers exist for making the enrollment and certification process simple?
   4. What is your process for following up with people who fill out an application form for assistance?
   5. Do you have a sense of how your clients become aware of their WIC eligibility?
   6. Are there certain areas where you find individuals have misconceptions of how the program works?
   7. Are there specific state or federal policies that are creating barriers?
3. If resources and time were not a constraint, what changes would you make to facilitate greater enrollment and utilization of WIC services and food benefits?
4. Often, individuals on WIC don’t end up using their benefits, or they stop as their children get older. Why do you think that is?
5. What could the healthcare community do to help with enrollment and retention in the NH WIC Program?
6. What is your relationship like with healthcare providers in your service area?
   1. What could improve these relationships?
7. In relationship to screening and referrals to other social services: What are your experiences like with sharing data across organizations in your community?
   1. What has worked well?
   2. Where have challenges occurred? How were they navigated?
   3. Do you have any direct, closed loop referral systems at your clinic for directly enrolling people into WIC? (tell me about them)
8. Is there anything else you would like to share?
9. Would it be okay to reach back out to you in the future?

**Interview Guide: Vermont WIC Staff**

*Thank you for agreeing to chat with me today. As we mentioned when we reached out, we are trying to develop community-healthcare partnerships to improve participation in the WIC Program [a federally funded program that supports the nutritional needs of women, infant, and children.].  The hope from these interviews is to get a better sense of what is working, and where there are challenges and opportunities for improvement. In particular, we know that many individuals who are eligible for WIC don’t sign up, and even among those who do sign up, many do not continue to attend appointments to receive benefits for their full eligibility period or make full use of their food benefits. Given your role with WIC, we are hoping you will share your expertise, experiences, and perspectives with us. This interview is completely confidential – none of your responses will be linked to your name. We can also skip any questions you prefer not to answer. Is now still a good time for you?  And do you mind if I record this conversation?*

*[if yes to recording, should restate at beginning of recording that you are recording, e.g. “Thanks for agreeing to have this conversation recorded.”*

*VT WIC*

1. What is your role with WIC? How long have you worked for VT WIC?
2. What are you most proud of with the VT WIC program? What’s working well?
   1. What do you wish you could change or improve?
   2. What stands in the way?
   3. What has helped?
   4. Do you have a sense of how your clients become aware of their WIC eligibility?
3. If resources and time were not a constraint, what changes would you make to facilitate greater enrollment and utilization of WIC services and food benefits?
4. Often, individuals on the WIC program to stop using it as their children get older. Why do you think that is?
5. What could the healthcare community do to help with enrollment and retention in the WIC Program?
6. There is a lot of variability in WIC coverage rates across the country. Do you have any ideas about what may account for these differences (processes, population, state policies, or environment)? (i.e., 2021 NH overall coverage rate is 53.6% compared to VT at 71.6%, ME at 54.7% and MA at 61.2%)
7. What is your relationship like with healthcare providers in your service area?
   1. What could improve these relationships?
   2. Do you have any direct, closed loop referral systems at your clinic for directly enrolling people into WIC? (tell me about them)
      1. Do you share (with consent) any data for the purpose of not duplicating services?
8. In relationship to screening and referrals to other social services: What are your experiences like with sharing data across organizations in your community?
   1. What has worked well?
   2. Where have challenges occurred? How were they navigated?
9. What is your relationship like with other programs, such as SNAP, Medicaid or TANF?
10. Is there anything else you would like to share?
